# Understanding Chromium Slag Recycling with Sintering–Ironmaking Processes: Influence of Cr_2_O_3_ on the Sinter Microstructure and Mechanical Properties of the Silico–Ferrite of Calcium and Aluminum (SFCA)

**DOI:** 10.3390/molecules29102382

**Published:** 2024-05-18

**Authors:** Ju Xu, Guojun Ma, Mengke Liu, Xiang Zhang, Dingli Zheng, Tianyu Du, Yanheng Luo, Wei Zhang

**Affiliations:** 1 The State Key Laboratory of Refractories and Metallurgy, Wuhan University of Science and Technology, Wuhan 430081, China; xuju@wust.edu.cn (J.X.); gma@wust.edu.cn (G.M.); zx91@wust.edu.cn (X.Z.); dinglizheng@wust.edu.cn (D.Z.); dty2021@wust.edu.cn (T.D.); 202102704170@wust.edu.cn (Y.L.); wei_zhang@wust.edu.cn (W.Z.); 2 Joint International Research Laboratory of Refractories and Metallurgy, Ministry of Education, Wuhan University of Science and Technology, Wuhan 430081, China; 3 Key Laboratory for Ferrous Metallurgy and Resources Utilization of Ministry of Education, Wuhan University of Science and Technology, Wuhan 430081, China

**Keywords:** Cr_2_O_3_, sinter, SFCA, first principles, mechanical property

## Abstract

Chromium slag is a solid waste of chromium salt production, which contains highly toxic Cr(VI) and significant amounts of valuable metals, such as Fe and Cr. Recycling chromium slag as a raw sintering material in sintering–ironmaking processes can simultaneously reduce toxic Cr(VI) and recover valuable metals. A micro-sintering experiment, compressive strength test, microhardness test, and first-principles calculation are performed to investigate the influence of Cr_2_O_3_ on the sintering microstructure and mechanical properties of the silico-ferrite of calcium and aluminum (SFCA) in order to understand the basis of the sintering process with chromium slag addition. The results show that the microstructure of SFCA changes from blocky to interwoven, with further increasing Cr_2_O_3_ content from 0 wt% to 3 wt%, and transforms to blocky with Cr_2_O_3_ content increasing to 5 wt%. Cr_2_O_3_ reacts with Fe_2_O_3_ to form (Fe_1−*x*_Cr*_x_*)_2_O_3_ (0 ≤ *x* ≤ 1), which participates in forming SFCA. With the increase in Cr doping concentrations, the hardness of SFCA first decreases and then increases, and the toughness increases. When Cr_2_O_3_ content increases from 0 wt% to 3 wt%, the SFCA microhardness decreases and the compressive strength of the sintered sample increases. Further increasing Cr_2_O_3_ contents to 5 wt%, the SFCA microhardness increases, and the compressive strength of sintered sample decreases.

## 1. Introduction

Chromium slag is a solid by-product of chromium salt manufacture in the chemical industry [1,2]. It is recognized as one of the hazardous solid wastes due to the fact that it contains harmful components, such as carcinogenic calcium chromate and highly toxic water-soluble Cr(VI) [3,4,5]. The accumulation and landfills of chromium slag, therefore, usually cause serious pollution to soil and groundwater, which severely endangers the ecological environment and human health [6,7]. It is estimated that the cumulative accumulation of chromium slag in China has exceeded 6 million tons [8,9].

At present, chromium slag is treated by reduction, stabilization, and comprehensive utilization processes around the world [10,11,12,13,14]. Within these treatment processes, secondary wastes are generated that cause environmental pollution. Simultaneously, the precious metals in chromium slag cannot be reused. Furthermore, the low utilization rate of chromium slag is one of the reasons for the accumulation problem of chromium slag. The chemical composition of chromium slag is mainly composed of Fe_2_O_3_, Al_2_O_3_, SiO_2_, Cr_2_O_3_, and MgO, which makes it similar to iron ore. Therefore, to improve the economic value and utilization efficiency of chromium slag, applying the chromium slag as a raw sintering material is a successful method. It can not only reduce Cr(VI) in chromium slag under a reducing atmosphere to realize the harmless utilization of chromium slag but also efficiently recover valuable metal components, such as Fe and Cr [15].

The sinter is an intermediate product that will eventually be used as a raw material in the blast furnace ironmaking process; hence, the operation of blast furnace smelting will depend on the quality of the sinter [16,17]. Previous studies demonstrated that adding chromium slag to the sintering raw materials deteriorated the tumble strength of the high-basicity sinter, which had a notable influence on the quality of the high-basicity sinter [18]. The drum strength of the sinter mainly depends on the microstructure of the sinter and the self-strength of the binder phase in the sinter. However, the silico-ferrite of calcium and aluminum (SFCA) is the primary bonding phase of the high-basicity sinter [19].

Several works found that Fe_2_O_3_, Al_2_O_3_, SiO_2_, and MgO played a remarkable role in the mineral microstructure of the sinters and the mechanical properties of SFCA in sinters [20]. Gan et al. [21] and Higuchi et al. [22] found that the fraction of SFCA increased along with the amount of Al_2_O_3_ in the sinter, increasing the sinter’s mechanical strength. When the Al_2_O_3_ content is above 1.8%, weak flake SFCA would form, reducing the mechanical strength of the sinter. Shi et al. [23] and Zhou et al. [24] found that with the increase in MgO content, the formation of MgO·Fe_2_O_3_ by the reaction between MgO and Fe_2_O_3_ increased the formation temperature of the liquid phase in the sinter, resulting in a decrease in the liquid phase content and calcium ferrite mass fraction, which leads to a decrease in the strength of the sinter. Wang et al. [25,26] found that increasing the SiO_2_ content in the sinter to 4 wt% promoted the formation of calcium ferrite. Further increasing the SiO_2_ content caused the formation of silicate, which reduced the contact surface between CaO and Fe_2_O_3_, inhibited the formation of calcium ferrite, and reduced the strength of the sinter. Xue et al. [27,28] studied the influence and mechanism of basicity and temperature on the bonding phase strength (BS) and microstructure of high-chromium vanadium–titanium magnetite (HCVTM). The results showed that the BS of HCVTM sintered increased slightly with increasing temperature because the increase in temperature was beneficial to the formation of SFCA. Tu et al. [29] made composite pellets of chromium slag and carbon-containing dust for co-sintering with limonitic laterite. The results showed that the addition of more than 20% of chromium slag destroyed the drum strength of the sinter. Cr(III) mainly existed in spinel and SFCA. However, the effect of Cr_2_O_3_ content on the microstructure of the sinter, as well as the self-strength of SFCA, has not been reported yet.

The advancement of simulation technology in recent years has promoted the research of material toughening and strengthening mechanisms at the microscale and created a connection between theory and experiment [30]. The first-principles approach involves analyzing the properties of materials at the molecular and electron level, such as mechanical properties and electronic structure, thereby enabling the prediction of material performance to a certain extent [31]. In the field of materials, first principles have been widely used to predict the properties of iron, steel, and alloys [32]. First-principles calculations can provide theoretical guidance for alloy composition design [33]. This research method holds significant potential for practical applications in the steel industry. Therefore, it is beneficial to analyze the mechanical properties of SFCA at the microlevel by applying the first-principles calculation method. First-principles calculations can be used to determine the bulk modulus, shear modulus, Young’s modulus, Poisson’s, and Pugh’s modulus ratios of SFCA crystals. These parameters can predict the strength and toughness of SFCA. Yang et al. [34] investigated the impact of Al doping on the mechanical properties of calcium ferrite crystal via first-principles calculations. The results demonstrated that Al doping can increase the crystal strength of calcium ferrite.

In this work, the effects of Cr_2_O_3_ on the strength of sinters and the microhardness of SFCA in sinters were investigated via compressive strength and microhardness tests. The composition and morphology of the phases in the sinter were detected via X-ray diffraction (XRD) and scanning electron microscopy using an energy-dispersive spectrometer (SEM-EDS). The influence of Cr doping on the mechanical properties of SFCA was revealed via first-principles calculation. The aim of this study is to provide theoretical guidance and an experimental basis for the utilization of chromium slag in the sintering process.

## 2. Results and Discussion

### 2.1. Mineral Composition and Microstructure of the Sinter

#### 2.1.1. Mineral Composition of the Sinter

**Figure 1:**
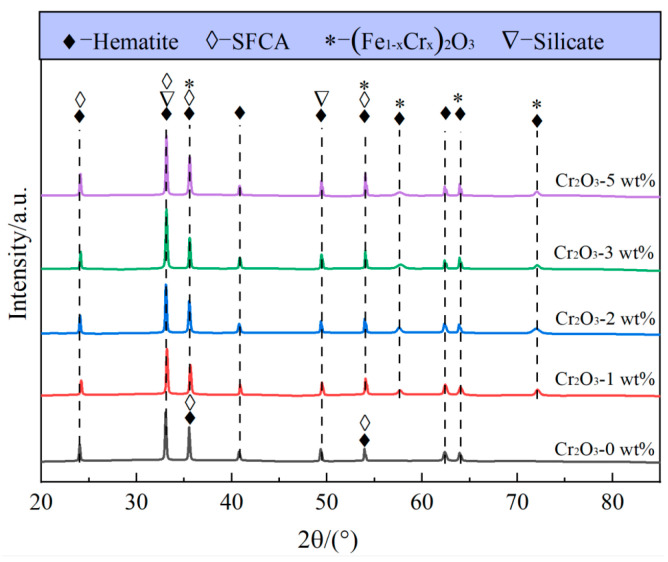
XRD patterns of samples with different Cr_2_O_3_ contents.

**Figure 2:**
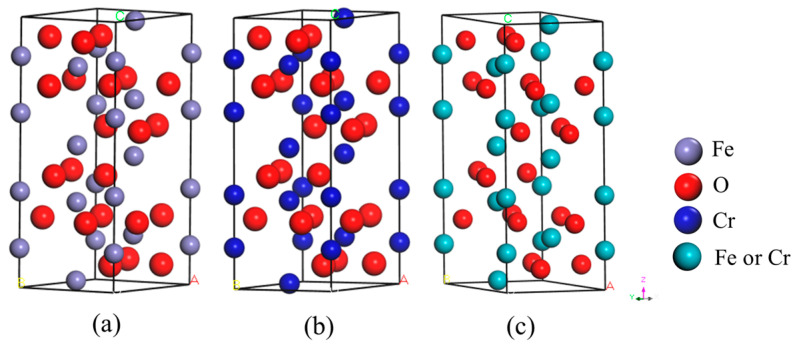
Crystal structures of Fe_2_O_3_, Cr_2_O_3_ and (Fe_1−*x*_Cr*_x_*)_2_O_3_: (**a**) Fe_2_O_3_; (**b**) Cr_2_O_3_; (**c**) (Fe_1−*x*_Cr*_x_*)_2_O_3_.

Figure 1 displays the XRD patterns of sinters with different Cr_2_O_3_ contents. It can be seen that the main phases in the specimens are hematite (Fe_2_O_3_), the silico-ferrite of calcium and aluminum (SFCA), and silicate. When the Cr_2_O_3_ content in sinters increases to 1 wt%, the diffraction peaks corresponding to (Fe_1−*x*_Cr*_x_*)_2_O_3_ (0 ≤ *x* ≤ 1) can be observed in the XRD pattern, and the intensity increases with the increase in Cr_2_O_3_ content. Figure 2 indicates the crystal structures of Fe_2_O_3_, Cr_2_O_3_, and (Fe_1−*x*_Cr*_x_*)_2_O_3_. It is clear that the crystal structures of Fe_2_O_3_ and Cr_2_O_3_ both belong to the monoclinic crystal system. The ionic radii of Fe and Cr are 0.55 Å and 0.62 Å, respectively. The insignificant difference between these ionic radii provides the conditions for Fe_2_O_3_ and Cr_2_O_3_ to form continuous solid solutions [35]. Therefore, Cr on the surface of hematite Fe_2_O_3_ easily migrates into the lattice of Fe_2_O_3_ by diffusion, replaces Fe, and occupies the iron ion vacancies in the hematite lattice forming (Fe_1−*x*_Cr*_x_*)_2_O_3_.

#### 2.1.2. Microstructure of Sinters

**Figure 3:**
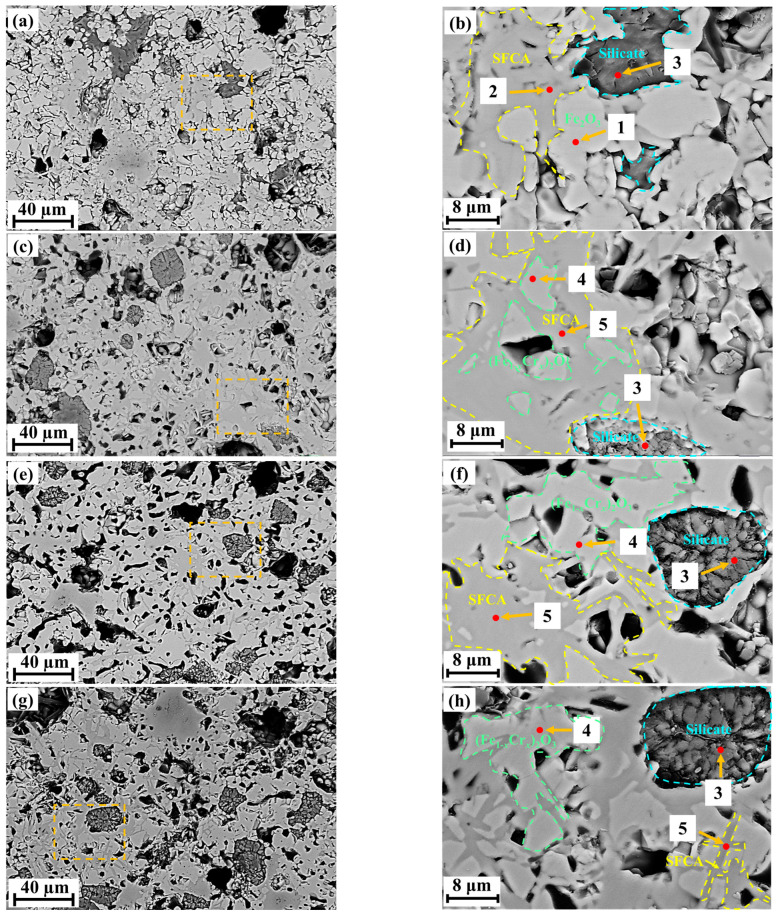

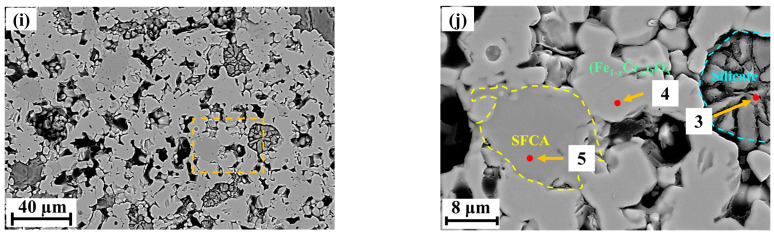
SEM results of samples with different Cr_2_O_3_ content. (**a**,**b**): 0 wt% Cr_2_O_3_; (**c**,**d**): 1 wt% Cr_2_O_3_; (**e**,**f**): 2 wt% Cr_2_O_3_; (**g**,**h**): 3 wt% Cr_2_O_3_; (**i**,**j**): 5 wt% Cr_2_O_3_; (**b**,**d**,**f**,**h**,**j**) are enlarged images of the enclosed areas of (**a**,**c**,**e**,**g**,**i**), respectively.

To reveal the influence of Cr_2_O_3_ on the microstructure of sinters, SEM-EDS was used to characterize the morphology and composition of phases in sintered samples. The results are shown in Figure 3 and Table 1, respectively. The sintered samples were discovered to include four phases, as demonstrated in the SEM results. These phases, when paired with the EDS analysis (Table 1), were identified as hematite, SFCA, silicate, and (Fe_1−*x*_Cr*_x_*)_2_O_3_, which is compatible with the XRD results (Figure 1).

Figure 3 exhibits the SEM results of sintered samples with different Cr_2_O_3_ contents. As is verified in Figure 3a,b, the dominant phases are hematite with an irregular shape, blocky SFCA, and massive silicate bonding hematite. The SFCA is unevenly distributed, and its size is large. When Cr_2_O_3_ content increases to 1.0 wt%, the dominant phase in the sintered sample is irregularly shaped (Fe_1−*x*_Cr*_x_*)_2_O_3_, and an interwoven SFCA appears, which is presented in Figure 3c,d. The distribution of interwoven SFCA is more uniform, while the content of plate SFCA is lower. When the content of Cr_2_O_3_ increases to 2.0 wt% and 3.0 wt%, the main phase in sintered samples is (Fe_1−*x*_Cr*_x_*)_2_O_3_, which is bonded by interwoven SFCA and massive silicate, as presented in Figure 3e,f and Figure 3g,h, respectively. The interwoven SFCA content gradually increases. Increasing the Cr_2_O_3_ content to 5.0 wt%, (Fe_1−*x*_Cr*_x_*)_2_O_3_ is the dominant phase, which is illustrated in Figure 3i,j. SFCA is shown as a block and its content decreases; the microstructure of the silicate phase remains constant, but its content increases.

It can be seen from the EDS results in Table 1 that in the sintered samples without Cr_2_O_3_, the hematite consists of Fe and O elements and the SFCA consists of Fe, Ca, Si, Al, and O elements. From the EDS results of point 4 in Figure 3d,f,h,j, a new phase can be observed in the sintered samples containing Cr_2_O_3_ and is identified to be (Fe_1−*x*_Cr*_x_*)_2_O_3_ which is composed of Fe, O, and Cr elements. All SFCA in the sintered samples containing Cr_2_O_3_ contain a small amount of Cr. Moreover, the atomic ratio of the Cr element in (Fe_1−*x*_Cr*_x_*)_2_O_3_ and SFCA increases with an increase in Cr_2_O_3_ content in sintered samples. This indicates that Cr elements participate in the formation of (Fe_1−*x*_Cr*_x_*)_2_O_3_ and SCFA during the sintering process.

**Figure 4:**
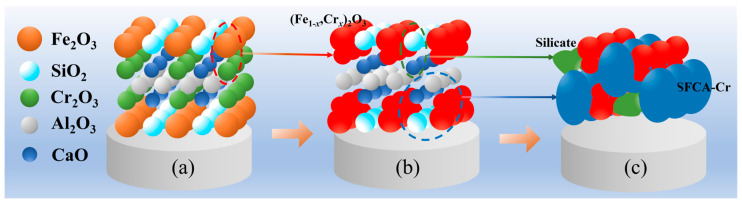
Formation mechanism of SFCA with Cr.

The reaction mechanism is schematically depicted in Figure 4. Fe_2_O_3_ and Cr_2_O_3_, shown in the marked red region in Figure 4a, react to form (Fe_1−*x*_Cr*_x_*)_2_O_3_, which is displayed as the red phase in Figure 4b. SiO_2_ and CaO in the sinter (in the marked green region in Figure 4b) react to form silicate. The formed silicate presents as the green phase in Figure 4c, which is the generation of silicate observed via SEM-EDS. In the sintered sample, (Fe_1−*x*_Cr*_x_*)_2_O_3_ reacts with SiO_2_, Al_2_O_3_, and CaO to form Cr-containing SFCA, which exhibits the blue phase in Figure 4c. Therefore, some amounts of Cr elements are found in the SFCA in the sintered samples with Cr_2_O_3_. The formation temperature of (Fe_1−*x*_Cr*_x_*)_2_O_3_ is about 500 °C, while the formation temperature of SFCA is 1107 °C–1164 °C [36]. (Fe_1−*x*_Cr*_x_*)_2_O_3_ is formed first during the sintering process, and then the formed (Fe_1−*x*_Cr*_x_*)_2_O_3_ reacts with CaO, SiO_2,_ and Al_2_O_3_ to generate the SFCA.

### 2.2. Mineral Composition of Sinter

The aforementioned results found that with the increase in Cr_2_O_3_ content in the sintered samples, Cr atoms were gradually doped into SFCA crystals, which could affect the mechanical properties of SFCA and thus the metallurgical quality of the sinter ore. Therefore, the first-principles calculation was conducted to reveal the influencing mechanism of Cr doping into SFCA crystals on the mechanical properties of SFCA crystals. First, the crystal structure of SFCA crystals with and without Cr doping was geometrically optimized via the quantum mechanics program CASTEP [37]. Then, the elastic constants were calculated via the stress–strain method based on the optimized structure.

Table 2 lists the calculated lattice constants of SFCA crystals with different amounts of Cr doping. It can be found that adding Cr to SFCA crystals results in obvious lattice deformation. Furthermore, with an increase in doped Cr concentrations, the cell volume rises while the lattice remains unchanged. The radii of Fe and Cr atoms are 1.56 Å and 1.66 Å, respectively, while the radii of their covalent bonds are 1.16 Å and 1.22 Å, respectively. When Cr atoms replace Fe atoms, the small atomic radii are replaced by large atomic radii. Simultaneously, the small covalent bond radii are replaced by a large covalent bond radius, so the volume of SFCA crystals increases with the increase in the amount of Cr atoms.

To investigate the effect of doped Cr concentrations on the mechanical properties of SFCA crystals, the bulk modulus and shear modulus of SFCA crystals with different doped Cr concentrations are analyzed. To ensure that the calculated data are valid, the stability of the crystal mechanics of Cr-doped SFCA crystals should be investigated before considering the elastic property. The elastic constants of these structures satisfy the Born stability criterion for trigonal crystalline systems [38,39]. The sixth-order symmetric matrix C must be positive definite, meaning that all primary determinants must be greater than zero, as this is a sufficient condition for the stability of the triclinic system. The calculated elastic constants of SFCA crystals with different doped Cr concentrations are listed in Table 3. This indicates that the SFCA crystals with doped Cr concentrations from 0 at.% to 4.4 at.% are elastically stable.

**Figure 5:**
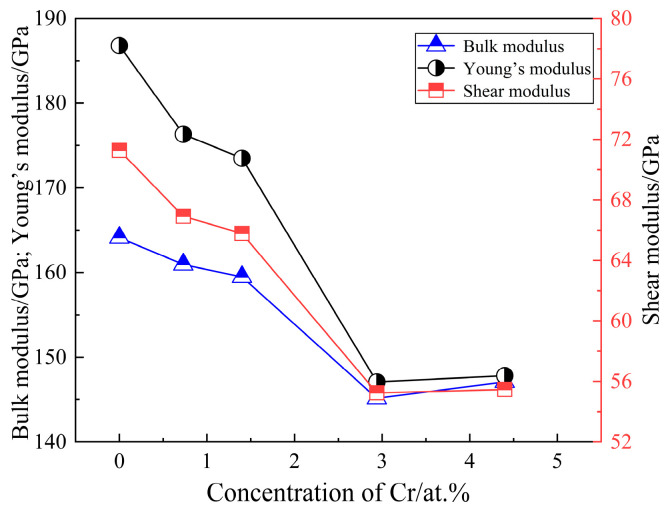
Bulk modulus, shear modulus, and Young’s modulus for SFCA crystals with different doped Cr concentrations.

Figure 5 illustrates the calculated results of the bulk modulus, shear modulus, and Young’s modulus of SFCA crystals with different doped Cr concentrations. The bulk modulus, shear modulus, and Young’s modulus of SFCA crystals decreased initially and subsequently increased when the Cr doping concentration rose from 0 at.% to 4.4 at.%. The bulk modulus, shear modulus, and Young’s modulus of SFCA crystal decrease from 164.112 GPa to 145.139 GPa, 71.276 GPa to 55.247 GPa, and 186.786 GPa to 147.079 GPa, with increasing Cr doping concentrations from 0 at.% to 2.94 at.%, respectively. The bulk modulus, shear modulus, and Young’s modulus of SFCA crystals increase to 147.097 GPa, 55.464 GPa, and 147.814 GPa with a further increase in Cr doping concentrations to 4.4 at.%, respectively.

There exists a monotonic relationship between the hardness of SFCA and the elastic constant C44: the higher the C44, the greater the hardness of SFCA [40]. As listed in Table 3, C44 exhibits a decreasing trend followed by an increasing trend. As the Cr doping concentration increases from 0 at.% to 2.94 at.%, the value of C44 decreases from 77.589 GPa to 55.212 GPa. Similarly, as the Cr doping concentration increases from 2.94 at.% to 4.4at.%, the value of C44 increases from 55.212 GPa to 59.503 GPa.

**Figure 6:**
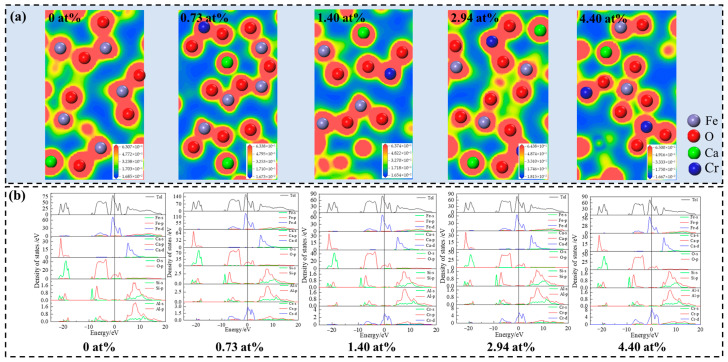
(**a**) The electron density distribution of SFCA crystals doped with different doped Cr concentrations; (**b**) the density of states of SFCA crystals doped with different doped Cr concentrations.

The variation in hardness is the result of the combined effects of multiple factors, including atomic radius, interatomic charge density, and lattice defects [41]. It can be seen from Table 2 that the atomic radius of Cr is larger than that of Fe, which leads to the expansion of the lattice volume of the SFCA crystal when Cr atoms replace Fe atoms, resulting in internal defects that reduce the mechanical properties of crystals [42,43]. Furthermore, doping can change the electron density and impact the strength of atom bonds, resulting in various mechanical characteristics [40]. The electron density distribution and density of states of SFCA crystals doped with different doped Cr concentrations are indicated in Figure 6. It can be seen from Figure 6a that the electron density distributed between Cr and O atoms increases as the doping amount of Cr atoms increases. The doping of Cr atoms causes a considerable buildup of overlapping electrons between the Cr and O atoms, resulting in the formation of potent covalent bonds that increase crystal hardness. Meanwhile, in the interval from −9 to 3 eV, the d orbital of Fe, the p orbital of O, and the d orbitals of Cr generate strong hybridization, as depicted in Figure 6b. The hybridization of electrons is the main factor that distinguishes the hardness of materials and can be used to obtain the hardness enhancement of the SFCA crystals after Cr doping. Hence, when the doping concentration of Cr is within the range of 0–2.94 at.%, the increase in lattice volume, which results in severe internal defects, is the primary reason for the drop in the hardness of SFCA crystals. However, as the Cr doping concentration increases to 4.4 at.%, more Cr atoms participate in strong covalent interactions with O atoms, increasing the crystal’s hardness. Therefore, from any perspective, it can be concluded that the hardness of SFCA decreases as the doping concentration of Cr increases from 0 at.% to 2.94 at.% and then increases as the doping concentration of Cr increases from 2.94 at.% to 4.4 at.%.

**Figure 7:**
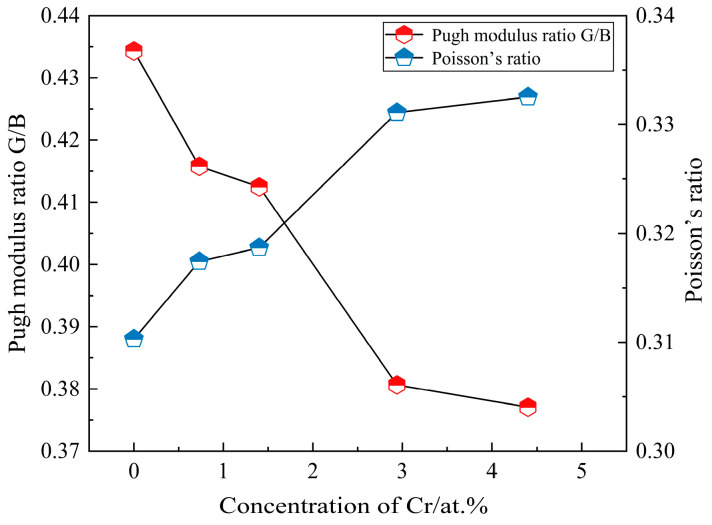
G/B values and Poisson’s ratio for SFCA crystals doping with different Cr concentrations.

Pugh’s modulus ratio G/B and Poisson’s ratio υ are important parameters for evaluating the toughness and brittleness of materials. A larger value of G/B indicates the increased brittleness of the material with a critical value of 0.571, while a larger Poisson’s ratio indicates a material with better toughness [44,45]. Figure 7 reveals the calculated results of the Pugh modulus ratio G/B and Poisson’s ratio υ of SFCA crystals with various doped Cr concentrations. It is clear that all G/B values of SFCA crystals with various doped Cr concentrations are less than 0.571, indicating that the crystal structure is ductile both before and after doping. When the doped Cr concentration increases from 0 at.% to 4.4 at.%, the G/B value decreases from 0.434 to 0.381, and Poisson’s ratio increases from 0.310 to 0.331. This demonstrates that the addition of Cr can enhance the toughness of SFCA crystals. SFCA with better toughness can absorb more energy from the impact or dynamic load, which can bear larger deformations.

### 2.3. Mineral Composition and Microstructure of the Sinter

#### 2.3.1. Effect of Cr_2_O_3_ on Microhardness of SFCA

**Figure 8:**
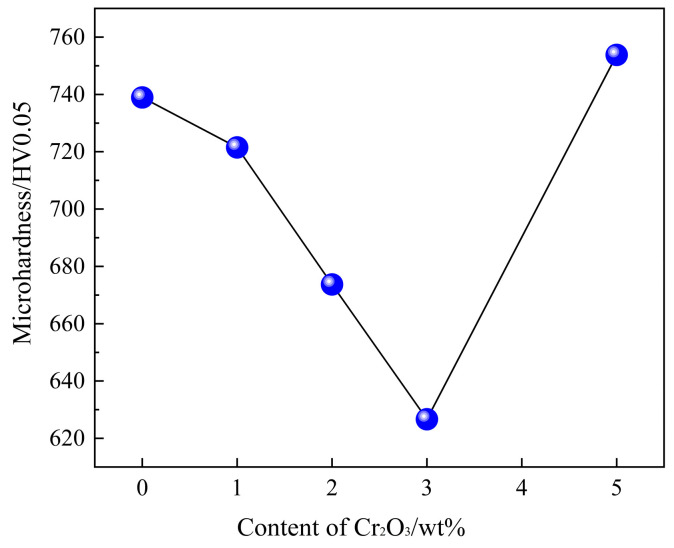
Effect of Cr_2_O_3_ content on microhardness of SFCA.

Figure 8 displays the effect of Cr_2_O_3_ content on the microhardness of SFCA. It can be observed from Figure 8 that with the increase in Cr_2_O_3_ content, the microhardness of SFCA shows a trend of first decreasing and then increasing. When the Cr_2_O_3_ content increases from 0 wt% to 3 wt%, the microhardness of SFCA decreases from 738.94 HV0.05 to 626.67 HV0.05. With a further increase in the Cr_2_O_3_ content to 5 wt%, the microhardness of SFCA increases to 753.77 HV0.05.

The mechanical properties of materials exhibit a certain connection at both the microscopic and macroscopic levels. On the macroscopic scale, the material’s microhardness is directly proportional to the microscopic aspects of the crystal’s bulk modulus, shear modulus, and Young’s modulus [46]. According to first-principles calculations, it was observed that the hardness of SFCA crystals decreased, and toughness increased as the Cr doping concentration escalated from 0% to 2.94%. Conversely, an increase in Cr doping concentration from 2.94% to 4.4% resulted in an elevation in the hardness of SFCA crystals and a decrease in toughness. These align with the experimental results obtained from microhardness tests conducted on the SFCA phase in the samples. The first-principles simulation lacks consideration of the simultaneous substitution of Fe and Al atoms for Cr atoms. However, this represents a noteworthy aspect that will be subjected to a more comprehensive study in the future. The forthcoming research aims to provide a deeper understanding of the atomic interactions and behaviors within the system under investigation, with a focus on elucidating the effects of the substitution process on the properties of the material.

#### 2.3.2. Effect of Cr_2_O_3_ on the Compressive Strength of Sinter

**Figure 9:**
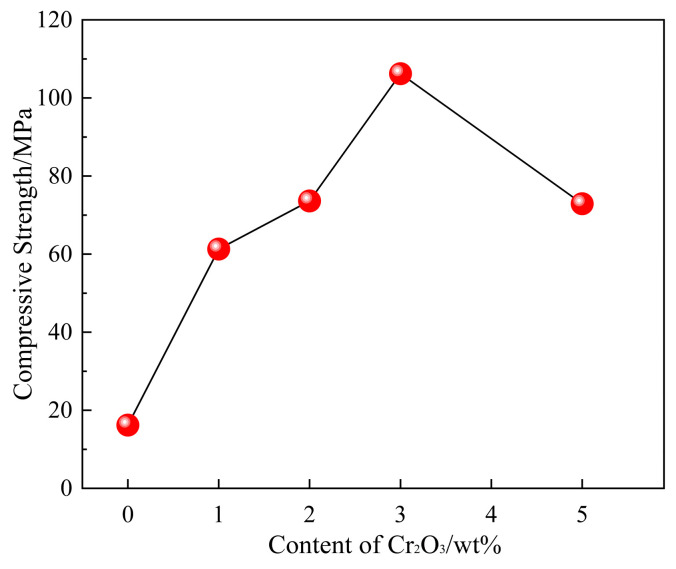
Effect of Cr_2_O_3_ content on the compressive strength of the sinter.

Figure 9 indicates the effect of Cr_2_O_3_ content on the compressive strength of the sinter. It is clear in Figure 9 that the compressive strength of the sinter without Cr_2_O_3_ addition is 16.2 MPa. When the Cr_2_O_3_ content increases to 3 wt%, the compressive strength of the sample increases to 106.2 MPa. While the compressive strength of sintered samples decreases to 72.9 MPa with a further increase in Cr_2_O_3_ content to 5 wt%. The results demonstrate that adding Cr_2_O_3_ can improve the compressive strength of the sintered sample, and excessive Cr_2_O_3_ deteriorates the compressive strength of the sintered sample.

Many factors affect the strength of sintered samples, such as the strength and structure of SFCA and the formation of the liquid phase in sintered samples. Although the microhardness of SFCA in sintered samples decreases with an increase in Cr_2_O_3_ content, the formation of more interwoven SFCA can effectively inhibit crack propagation, which makes the compressive strength of interwoven SFCA better than that of a single blocky SFCA and enhances the compressive strength of sintered samples [20,47,48]. When Cr_2_O_3_ content increases to 5 wt%, more Cr participates in Fe_2_O_3_, which enhances the stability of (Fe_1−*x*_Cr*_x_*)_2_O_3_. It inhibits the reaction between (Fe_1−*x*_Cr*_x_*)_2_O_3_ and CaO, Al_2_O_3_, and SiO_2_ to form SFCA and leads to a reduction in the amount of liquid phase in sintered samples, which results in a decrease in the compressive strength of the sinter ore.

## 3. Experimental Section

### 3.1. Sample Preparation

In order to eliminate the impact of minor components in chromium slag, analytical grades of (AR) Fe_2_O_3_ (≥99.9 wt%), SiO_2_ (≥99.9 wt%), Al_2_O_3_ (≥99.9 wt%), CaO (≥96.0 wt%), and Cr_2_O_3_ (≥99.9 wt%) were used as raw materials to generate experimental sintered samples. The raw material ratios in this experiment were designed based on the basicity and composition of the sintered ore when chromium slag was used as a partially sintering raw material, and the basicity *R* (CaO/SiO_2_) of the sintered ore was designed to be 1.8 [49]. The Cr_2_O_3_ contents were 0 wt%, 1 wt%, 2 wt%, 3 wt%, and 5 wt%, respectively, and the mass ratio of other components maintained constant. The chemical compositions of the samples are listed in Table 4.

**Figure 10:**
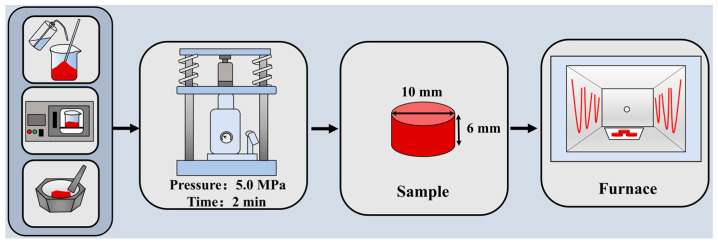
Process schematic of sample preparation and sintering.

According to the designed chemical compositions of the sintered samples, as depicted in Table 4, the raw materials were mixed with anhydrous ethanol for 30 min and dried at 100 °C for 2 h. The dried raw materials are ground and mixed thoroughly in an agate mortar. Then, about 1.5 g of raw material was pressed in a cylindric mold with the size of *Φ*10 mm × 6 mm under the pressure of 5.0 MPa and held for 2 min. To simulate the sintering process, the cylindric samples were maintained at 1200 °C for 10 h in the electric resistance furnace under an air atmosphere and then cooled to room temperature in the furnace. The process schematic of sample preparation and sintering is illustrated in Figure 10.

### 3.2. Characterization of Sintered Samples

The sintered samples were ground into powders to determine the crystalline phases via XRD with Cu-Kα radiation (X’Pert PRO MPD, PANalytical, Almelo, The Netherlands). Parts of the sintered samples were inlaid with epoxy resin and polished, followed by coating a thin gold film onto the polished sample to enhance the electric conductivity of the sample. The microhardness of the SFCA phase in each sample was measured using an image-processing microhardness tester (HV-1000A, Shen Youda industrial, Shanghai, China). The morphology and chemical composition of phases in the sintered samples were identified via SEM-EDS (Nova NanoSEM400, FEI Hillsboro USA, EVO 010, Zeiss, X-MaxN 79416, Oxford UK). The compressive strength of samples was tested by a flexural and compressive testing machine (SJY-II, Xiangtan Instrument, Xiangtan, China), and the compressive strength was the average value of ten tests.

### 3.3. Method and Model of First-Principles Calculation

Based on the density functional theory (DFT), the calculations were performed using the CASTEP quantum mechanics module in the Materials Studio software. As Fe and Cr elements are both transition metal elements, their spin polarization was considered in the calculations. The valence electron configurations of Fe, Ca, Cr, O, Si, and Al in SFCA are 3d^6^4s^2^, 4s^2^, 3d^5^4s^1^, 2s^2^2p^4^, 3s^2^3p^2^, and 3s^2^3p, respectively. The generalized gradient approximation (GGA) was employed to optimize SFCA cells and calculate the elastic modulus. The PBE generalized function was chosen for the electron correlation. The plane wave phase energy and k-point grid were chosen to be 450 eV and [2 × 2 × 2], respectively. The wave phase energy and k-point grid of 2 × 1 × 1 supercell was chosen to be 450 eV and [2 × 2 × 1], respectively.

To investigate the effect of Cr doping on the mechanical properties of SFCA crystals, a certain concentration of Fe atoms in the SFCA crystals was randomly replaced with Cr atoms. The elastic modulus of different crystals was calculated via the CASTEP module. The bulk modulus and shear modulus of crystals were calculated using the Voigt–Reuss–Hill method. The Young’s modulus and Poisson’s ratio of crystals were calculated using Equations (1) and (2) [50,51,52]. Among them, the bulk modulus represents the resistance of the material to external homogeneous compression in the elastic regime. The shear modulus is the ratio of shear stress to shear strain, which characterizes the ability of material to resist the shear strain. Young’s modulus characterizes the ability of a solid material to resist deformation:

(1) $$E=\frac{9B\times G}{3B+G}$$

(2) $$\upsilon =\frac{3B-2G}{2(3B+G)}$$

where *B* is the crystal bulk modulus, GPa; *G* is the shear modulus, GPa; *E* is Young’s modulus, GPa; and *υ* is Poisson’s ratio.

**Figure 11:**
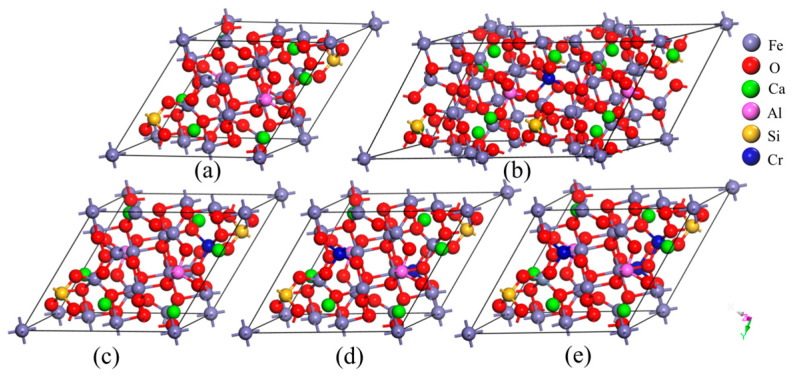
SFCA cells with different Cr doping amounts: (**a**) 0 at.%; (**b**) 0.73 at.%; (**c**) 1.40 at.%; (**d**) 2.94 at.%; (**e**) 4.40 at.%.

The crystal structure of SFCA was determined via the XRD results of sintered samples. The SFCA crystal [53] belongs to the trigonal crystal system with space group P-1, and each cell contained 18 Fe atoms, 6 Ca atoms, 2 Al atoms, 2 Si atoms, and 40 O atoms, as presented in Figure 11a. Figure 11b–e display the SFCA cells with doped Cr concentrations of 0.73 at.%, 1.4 at.%, 2.94 at.%, and 4.4 at.%, respectively. In order to accommodate the Cr doping concentration of 0.73 at.% in the SFCA cell, a 2 × 1 × 1 supercell needs to be created, as presented in Figure 11b. As the proportion of chromium atoms to all atoms in the crystal cell, the doped chromium concentration is calculated.

## 4. Conclusions

(1) The phases are Fe_2_O_3_, SFCA, and silicate in the sintered sample without Cr_2_O_3_. After adding Cr_2_O_3_ content (≤5 wt%) in sintered samples, (Fe_1−**x**_Cr*_x_*)_2_O_3_ is found, which results from the doping of Cr into Fe_2_O_3_. Furthermore, the Cr element is also found in the SFCA. With the addition of Cr_2_O_3_, the contents of Cr in (Fe_1−*x*_Cr*_x_*)_2_O_3_ and SFCA increase.

(2) The microstructure of the SFCA in sinters changes from being blocky to being interwoven, with an increase in the content of Cr_2_O_3_ from 0 wt% to 3 wt%, and then it transforms to being blocky with a further increase in the content of Cr_2_O_3_ to 5 wt%.

(3) When the doping concentration of Cr increases from 0 at.% to 4.4 at.%, the SFCA cell volume increases. The bulk modulus, shear modulus, and Young’s modulus decrease first and subsequently increase with the doping concentration of Cr, Pugh’s modulus ratio G/B decreases, and Poisson’s ratio increases. This demonstrates that the hardness of SFCA decreases as the doping concentration of Cr increases from 0 at.% to 2.94 at.%, and then it increases as the doping concentration of Cr increases from 2.94 at.% to 4.4 at.%. The toughness of SFCA increases with an increase in Cr doping concentrations.

(4) The microhardness of SFCA in sinters decreases from 738.94 HV0.05 to 626.67 HV0.05 with an increase in Cr_2_O_3_ content from 0 wt% to 3 wt%. The microhardness of SFCA increases to 753.77 HV0.05 with a further increase in the Cr_2_O_3_ content to 5 wt%. The variation in the microhardness of SFCA is consistent with the trend of first-principles calculations.

(5) The compressive strength of sintered samples increases from 12.6 MPa to 106.2 MPa with an increase in the content of Cr_2_O_3_ from 0 wt% to 3 wt% and then decreases to 72.9 MPa with a further increase in the content of Cr_2_O_3_ to 5 wt%.

## Figures and Tables

**Table 1:** Element content of sintered samples (at.%).

Location	Point	Fe	O	Ca	Si	Al	Cr	Phase
5b	1	50.5	49.5	-	-	-	-	Hematite
	2	31.0	54.0	8.4	3.1	3.5	-	SFCA
	3	-	58.6	20.8	20.5	-	-	Silicate
5d	4	53.1	45.9	-	-	-	1.0	(Fe_1−*x*_Cr*_x_*)_2_O_3_
	5	38.5	44.8	9.7	3.8	2.6	0.6	SFCA
	3	-	56.1	22.6	21.6	-	-	Silicate
5f	4	51.0	47.5	-	-	-	1.6	(Fe_1−*x*_Cr*_x_*)_2_O_3_
	5	42.9	49.7	4.4	1.6	2.0	0.9	SFCA
	3	-	58.5	27.0	13.7	-	-	Silicate
5h	4	49.3	48.5	-	-	-	2.2	(Fe_1−*x*_Cr*_x_*)_2_O_3_
	5	35.5	50.7	8.6	2.5	1.0	1.4	SFCA
	3	-	56.0	29.3	14.7	-	-	Silicate
5j	4	49.7	47.1	-	-	-	3.2	(Fe_1−*x*_Cr*_x_*)_2_O_3_
	5	41.1	46.6	5.0	2.7	2.5	2.7	SFCA
	3	-	67.6	21.0	11.4	-	-	Silicate

**Table 2:** Lattice parameters of SFCA crystals.

Doped Cr Concentrations	*a*/Å	*b*/Å	*c*/Å	α/(°)	β/(°)	γ/(°)	*V*/Å^3^
0 at.%	8.824	9.830	10.560	60.157	74.223	66.567	726.213
0.73 at.%	17.656 *	9.816	10.578	60.229	74.16	66.68	1454.73 *
1.4 at.%	8.819	9.809	10.591	60.317	74.215	66.678	727.921
2.94 at.%	8.754	9.892	10.741	59.313	74.00	66.415	730.033
4.4 at.%	8.764	9.859	10.732	59.755	73.788	66.764	732.551

*: It should be noted that the lattice constant “*a*” with a Cr doping amount of 0.73 at% is larger because the SFCA crystal builds up 2 × 1 × 1 supercells during calculations.

**Table 3:** Elastic constants of SFCA crystals with different doped Cr concentrations (GPa).

Cij	0 at.%	0.73 at.%	1.4 at.%	2.94 at.%	4.4 at.%
C11	256.568	257.856	245.337	223.854	211.351
C22	270.272	268.028	263.525	218.938	230.516
C33	241.514	227.568	235.047	227.912	227.579
C44	77.589	66.428	61.900	55.212	59.503
C55	71.859	68.499	70.849	54.912	48.917
C66	75.784	68.652	69.669	50.645	58.813
C12	137.168	129.89	128.352	104.603	106.489
C13	108.344	111.985	111.342	104.872	110.914
C14	7.399	6.114	0.361	6.397	1.088
C15	−4.516	−3.137	−5.609	−5.878	−9.866
C16	−9.782	−1.456	−6.548	−9.726	−10.157
C23	116.799	110.810	112.913	109.953	114.901
C24	−7.115	−4.727	−15.987	3.942	−5.904
C25	4.668	7.505	5.113	−0.783	−6.277
C26	−24.12	14.140	−20.712	1.803	−4.272
C34	−1.612	−2.667	−8.373	0.779	−8.557
C35	−2.660	−4.662	−2.214	−0.390	−3.566
C36	−7.844	−5.225	−3.955	−3.450	−4.281
C45	−2.177	−1.191	−1.576	0.923	3.262
C46	1.519	4.522	3.213	1.010	−0.159
C56	3.151	0.641	−0.125	−0.912	0.663

**Table 4:** Chemical compositions of the studied sintered samples (wt%).

Samples	Fe_2_O_3_	Al_2_O_3_	CaO	SiO_2_	Cr_2_O_3_
S1	81.38	2.13	10.57	5.94	0
S2	80.57	2.11	10.49	5.83	1
S3	79.76	2.09	10.38	5.77	2
S4	78.97	2.07	10.26	5.70	3
S5	78.18	2.05	9.50	5.28	5

## Data Availability

Not applicable.
